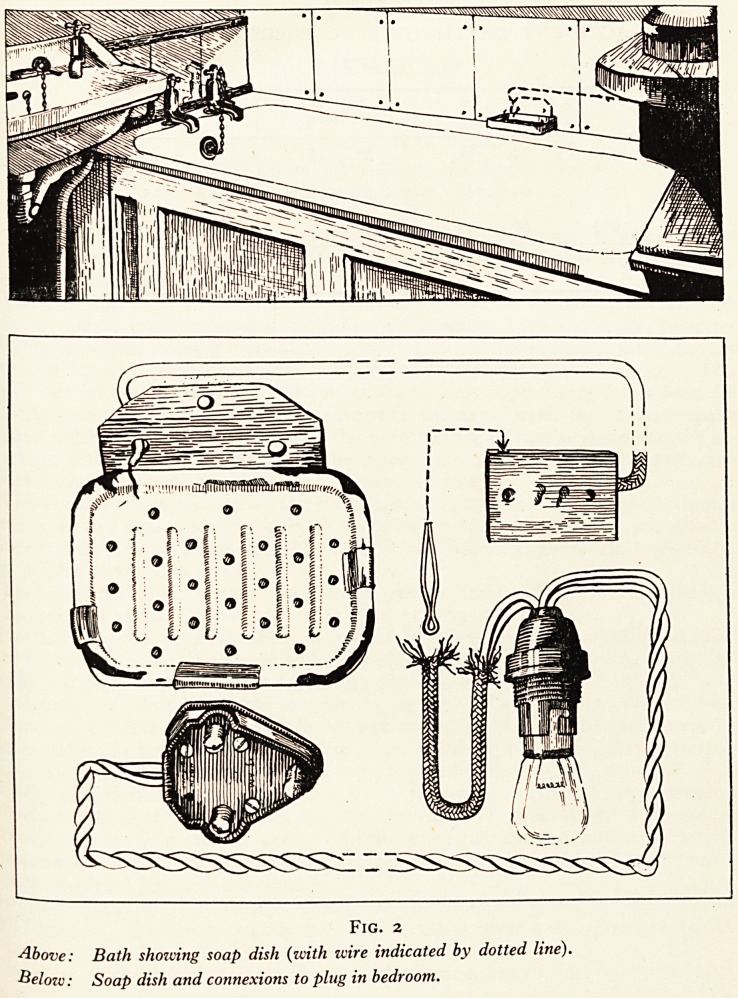# Domestic Deaths from Electrocution

**Published:** 1956-07

**Authors:** F. E. Camps

**Affiliations:** Department of Forensic Medicine the London Hospital Medical College


### DOMESTIC DEATHS FROM ELECTROCUTION

BY

F. E. CAMPS

*Department of Forensic Medicine the London Hospital Medical College*

Every year about a hundred people in England and Wales lose their lives by electrocution. Table I shows the total number of accidental deaths during the years 1942 to 1953, whilst Table II shows the number of deaths in 1952 and 1953 divided into those at work and those in the home. It is these deaths in the home that are considered in this paper.

When the wiring is done by an expert accidents are rare and most deaths are due to the ignorance and carelessness of the householder. Too often there is evidence of unsatisfactory wiring carried out by an amateur to effect replacements or improvements. Other common causes of accidents are neglect to obtain competent advice following " little shocks " from appliances, failure to repair damaged or worn insulation and attempts to carry out repairs without turning off the main switch.

The ordinary domestic electricity supply in England is at a pressure of about 240 volts, a figure which was agreed in 1946 and has been mandatory since 1947. Its strength, which is dependent upon the resistance between the terminals, will vary from 5 amperes for lighting to 30 amperes for heating and cooking points. Alternating current (A.C.) has almost completely replaced direct current (D.C.) and the standard is usually of 50 cycles per second. As a legacy of the past there are still in operation systems with non standard pressures ranging from 104–250 volts (A.C.) and others which operate at voltages of 100–240 volts (D.C.). The minimum current to kill from electrocution is stated to be about 65 milliamperes.

Death from electricity can occur in two ways, either by a sudden surprise shock causing vagal inhibition or by true electrocution which produces ventricular fibrillation or respiratory failure. Although the importance of the element of surprise should not be over emphasized, cases have been recorded in which death has occurred from touching a wire which was believed to be live but which was, in fact, dead.

From a practical point of view, it should be stressed that to receive the full effect it is necessary for a good " contact " to exist with " live " point and with " earth ". Dry human skin is a poor conductor with an average resistance of 2000–3000 ohms but when it is wet the skin resistance is reduced to as little as 500 ohms. If the skin is punctured the effective resistance will be that of the subcutaneous tissue, which is always very much lower than the skin. A current of about 12 milliamperes may make it impossible for a person to release his grip upon the " live object " if adequate " earth " is present. Other modifying factors will be the duration of exposure and the area of contact, whilst it is possible that the actual voltage received may be less than that computed because of electrical leakage.

It is a popular idea that water is a good conductor and therefore that a person is more vulnerable to electrocution when immersed in a bath. This is not always the case, for during the investigation of the case of R. *v.* Whybrow, which is mentioned later, experiments showed that a person sitting in that particular bath was, in fact, insulated. The bath itself is not the true reason for the bathroom being one of the two most " electrically " dangerous rooms in the house (the other is the kitchen) for the risk lies in the damp atmosphere when associated with a large number of metal objects

(mostly well " earthed ") such as the water taps and pipes, gas pipes and the like. For this reason it is necessary either to instal the electric switches outside the bathroom, or, if inside, to operate them with a pendant pull cord of non-conductive material. In spite of this safety precautions are rendered quite valueless by the introduction of some portable electrical apparatus such as an electric fire, a hair dryer, an electric kettle or even a wireless set. All, if properly constructed and intelligently used are safe enough but unfortunately they have often been made unsafe by unskilled wiring or rough handling. It is therefore no great surprise to hear of deaths from electrocution from time to time caused by foolhardy handling of electric appliances when seated in a bath.

When accidents occur, it is not uncommon for the electrical expert to find that an electric kettle or heater has been amateurishly rewired with omission of an earth lead (3rd wire) which makes it inevitable that, in the presence of a fault rendering the casing alive, any person handling it will take the full force of the current.

One early morning a husband heard a thud in the kitchen to which his wife had gone to make an early cup of tea. He got up at once and found his wife lying dead upon the kitchen floor. She had apparently been electrocuted from the casing of an electric kettle. This was confirmed by electric burns on the thumb and finger, whilst earth had occurred from her arm being in contact with one of the metal objects in the kitchen. The plug was examined by an expert electrician who found:

(1) There was no earth wire connected.

(2) That the person who had wired up the plug had left sufficient loose wire to flap over and short on to case. (Fig. 1).

Even a properly wired kettle may be dangerous if handled by an ignorant person as the following case will illustrate:

An elderly woman went out of her house one afternoon and forgot to switch off her electric kettle. This boiled dry during her absence and the safety mechanism blew out the plug making it impossible for her to replace the plug after refilling the kettle. Under the impression that there was "something stuck" she took the plug in one hand, thereby earthing herself with the earth safety metal strip upon it, and with the other hand introduced a metal screw driver into the positive terminal which was still connected "to a 25-ampere power plug at 'on', she somewhat naturally received the full charge with fatal results".

Careless maintenance of electrical equipment may well result in death and it is most surprising that many housewives of more than average intelligence will continue to use vacuum cleaners whose flex is bare from constant wear and tear. Recently a similar accident occurred with an electric lawn mower.

Two other kinds of domestic electrical equipment deserve mention—the radio and television sets. The former have been the cause of fatalities and afford an excellent example of the casual manner adopted by the public towards electricity. How often has a person been heard to say " A funny thing happened this morning, I got a shock off my radio " and when asked what had been done about it given the reply " Nothing " ?

Fatal accidents have been recorded from television sets, in one case due to the " frame " coming in contact with a metal grill in front. The fault in this particular set was completely remedied.* Nevertheless, education of the public to take notice of small " shocks" and call in an expert electrician would pay dividends.

Children, having small fingers, may be electrocuted by " poking " them into the holes of the standard plug or alternatively using metal objects such as skewers or knives. The close proximity of excellent " earths " such as gas pipes makes the " amusement " even more dangerous. This risk can be reduced by fitting plugs with safety shutters. A somewhat similar type of accident occurred some years ago under the following circumstances.

* The company called in all sets and modified them.

FIG. 1

*Above:* *Kettle plug as found (note frayed wire and cover out of place—no earth wire).*
*Below:* *After dismantling (note bare loose wires).*

A child aged five years was playing in a hall closet. Suddenly it collapsed and a doctor who was called found it to be dead. Detailed examination showed electric burns on the tips of the right index and middle fingers, one of which had a round depressed area. The post-mortem findings internally were consistent with electrocution. Examination of the closet showed a porcelain fuse box with bare wires sunk into the top in gutters too small to allow access of an adult finger but large enough to admit that of a child's finger. The bare end of the wire exactly fitted the depression on the child's finger and evidence showed that the child had been standing bare-footed upon a gas pipe when collapse took place.

Other fatal accidents have occurred from children climbing up and touching overhead electric cables and on one occasion a boy scaled a pylon for the purpose of throwing two metal cups tied together with string over the cables carrying the grid across the Thames. A blinding flash was seen for some miles away and the body of the culprit was found at the base of the pylon with a " broken neck " and extensive electrical flash burns down the whole of one side of his body resembling crocodile skin.

A case was recently recorded in the press of a child electrocuting itself by chewing through a length of electric flex.

### EVIDENCE OF ELECTROCUTION

It has been already stated that deaths from electrocution may be due either to ventricular fibrillation or respiratory failure, unfortunately the post-mortem appearances of either are not peculiar to the effect of passage of an electric current, for they are quite commonly seen, for example, in deaths resulting from coronary insufficiency or cerebro-vascular catastrophes.

The proof that electrocution is the cause of death must depend upon the identification of an " electric " burn. This demands a careful scrutiny of the body surface of *everyone* who dies in close proximity to any electric "live point".

The appearance of an electric burn has been *described* as a hard blister with a central white parchment bleached area and a hyperaemic zone of surrounding skin. The identification and interpretation of this lesion may not be easy and there is also considerable doubt as to whether such an appearance may not also be produced by exposure to an electric current shortly after death. The actual appearance of the electric lesions will vary according to the firmness of the contact. Thus, if the contact is poor in pressure and area, there will be small pricked out " spark burns ". If a diffuse area is involved with moderately firm application there may be a shaped burn which after a few hours may even show charring, especially with high voltage currents. The exit lesion may be less obvious and not so characteristic. In true electrocution burns may be completely absent, and it should be borne in mind that this may occur where a person is grasping an object, such as a pistol grip drill, at the time it becomes live. Electric burns are usually more extensive than their appearance suggests, and later considerable sloughing may occur.

Histologically an appearance has been described of elongation of the basal cells and nuclei but unfortunately this is by no means consistent and can occur from thermal burns.

The two alternative forms of death have already been mentioned. Probably most domestic deaths are due to ventricular fibrillation and the post-mortem appearances closely resemble those due to coronary artery insufficiency. Those due to respiratory failure will show more pulmonary oedema and certainly occur when the current passes through the medulla oblongata.

Suicide by electrocution is uncommon and such cases as occur are, as might be expected, somewhat bizarre in character. On one occasion death was brought about by preparing a circuit from the domestic supply using rings on a finger of each hand to connect to a plug, whilst in another a complicated electrode was placed over the praecordium, the circuit being closed by a wire from the same plug being held in one hand.

The use of electricity for homicide is believed to be very rare but it may be that cases have passed unrecognized owing to lack of identification or even search for electric burns. The following case illustrates an attempt at murder and scrutiny of it will show how easily it could have been missed if the woman had died and no suspicion had existed.

R. *v.* WHYBROW (*Essex Assizes* 1952)

In this case the accused was a married man living with his wife and children near Southend-on-Sea. One evening his wife decided to take a bath and whilst she was doing this he was in a bedroom on the other side of the passage. Suddenly she cried out saying that she had received an electric shock when she had touched the soap dish. After a short interval he went to the bathroom and decrying the suggestion of a shock touched the soap dish to show that she was mistaken. At the time she was satisfied with his statement but the next morning decided to telephone the Electricity Company to ask them to call and examine the soap dish. They were too busy to do this immediately and consequently she decided to examine it herself with the aid of her sister-in-law. After scraping away the plaster she found that it was fixed to the wall and that there was a wire to the bracket which had been made from a piece of tin by her husband. She traced the wire under the bath (boxed in) through the wall into the adjoining lavatory, where, after winding around the base of the lavatory pedestal, it entered the roof space above and then ran across the top to a cupboard in the bedroom where it was connected to a metal tube secured between two pieces of bakelite which were screwed to the wall. She then called the police who carried out a search and in a drawer found a length of india-rubber covered electric wire one end of which was attached to the positive terminal of a 15 ampere plug and the other to a large split pin which exactly fitted the metal tube in the cupboard. In the circuit had been introduced a 15-watt bulb with holder. (Fig. 2). A 15-ampere power-plug was fixed on the skirting board adjoining the cupboard and the whole circuit when connected allowed a current of 65 milliamperes to be detected at the soap dish (the reduction was due to the resistance of the bulb). Examination of the bath showed that a person seated in it would be completely insulated. This was due to the porcelain being intact, the runaway being insulated by the plaster which fixed it, leaving the only " earth " through the chain attached to the bath plug. An appreciable electric shock could be felt when the soap dish was touched.

Investigation showed that the husband was associating with a young girl without telling her parents that he was already married. His wife had discovered and resented this liaison. He had moreover been previously convicted of bigamy. His defence was that the apparatus was the earth of his wireless set but the jury found him guilty of attempted murder.

To summarize, the recognition of death from electrocution is not easy unless the medical practitioner who first sees the case is both suspicious in his approach and careful in his examination of the body. Two fatal cases of electrocution, although the possibility was considered and discarded at the time, were not detected until some weeks later. In neither case was there anything to suggest electrocution shown by autopsy and no local burns were present.

Examination of cases of electrocution which have occurred each year shows the main reason for the accidents is faulty wiring installed by amateurs.

The medical literature on electrocution is not extensive but valuable information on the subject can be found in the technical journals. The electricity authorities are always most courteous and helpful, and in this connexion I should like to express my thanks in particular to Mr. L. H. Jesty of the London Electricity Board for his helpful advice both in the preparation of this paper and in connexion with several cases I have met in practice.

### ADDITIONAL READING

*Legal Medicine* by R. B. H. Gradwohl—Chapter VIII (C. V. Mosby Co., St. Louis, 1954).
*Atlas Zurspurenkunde Der Elektrizitaet* by S. Jellninek (Springer, Vienna, 1955).

FIG. 2

*Above:* *Bath showing soap dish (with wire indicated by dotted line).*

*Below:* *Soap dish and connexions to plug in bedroom.*

TABLE I

ACCIDENT DEATHS FROM ELECTRIC CURRENT

| | 1942 | 1943 | 1944 | 1945 | 1946 | 1947 | 1948 | 1949 | 1950 | 1951 | 1952 | 1953 |
|---|---|---|---|---|---|---|---|---|---|---|---|---|
| Male | 88 | 94 | 79 | 81 | 90 | 78 | 77 | 68 | 69 | 85 | 94 | 86 |
| Female | 14 | 20 | 22 | 29 | 16 | 16 | 19 | 21 | 20 | 17 | 18 | 25 |

TABLE II

ACCIDENT DEATHS DUE TO ELECTRIC CURRENT
1952–53

| | *Total* | | *At work* | | *At home* | |
|---|---|---|---|---|---|---|
| | M. | F. | M. | F. | M. | F. |
| 1952 | 94 | 18 | 58 | 1 | 27 | 15 |
| 1953 | 86 | 25 | 56 | 3 | 13 | 22 |
| | 180 | 43 | 114 | 4 | 40 | 37 |

## Figures and Tables

**Fig. 1 f1:**
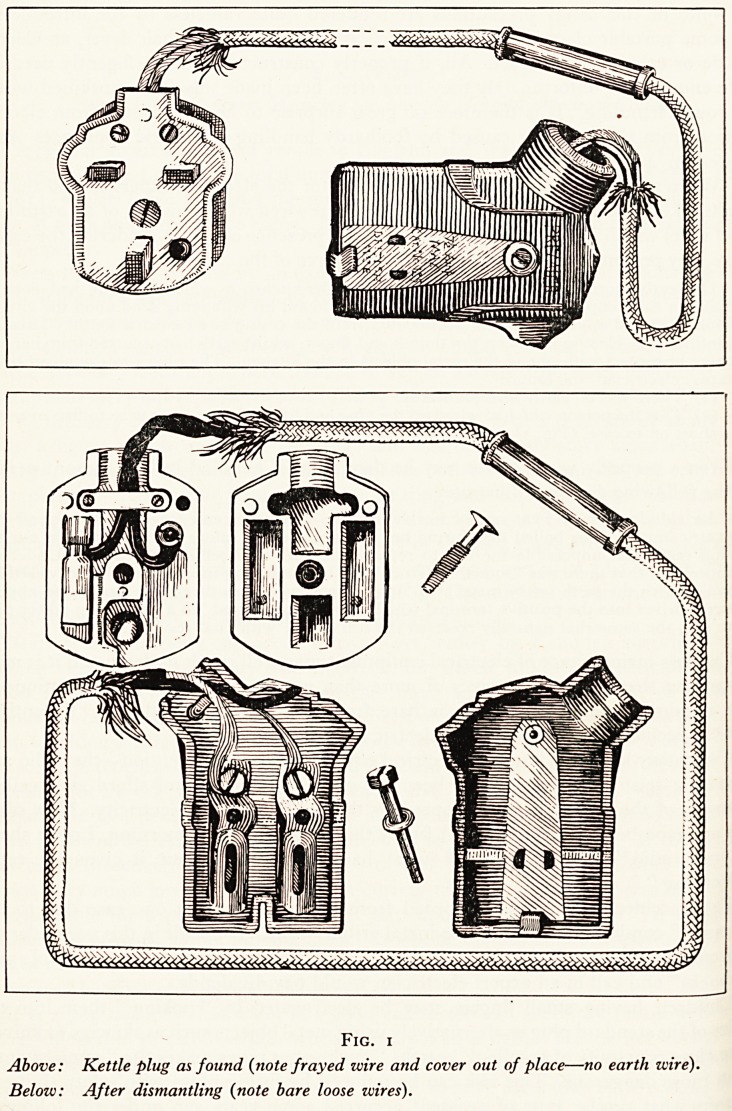


**Fig. 2 f2:**